# Co-infection does not predict disease signs in *Gopherus* tortoises

**DOI:** 10.1098/rsos.171003

**Published:** 2017-10-18

**Authors:** Chava L. Weitzman, Ryan Gov, Franziska C. Sandmeier, Sarah J. Snyder, C. Richard Tracy

**Affiliations:** 1 Ecology, Evolution, and Conservation Biology, University of Nevada, Reno, Reno, NV 89557, USA; 2 Department of Biology, University of Nevada, Reno, Reno, NV 89557, USA; 3 Department of Biology, Colorado State University, Pueblo, Pueblo, CO 81001, USA; 4 Science, Mathematics, and Computing, Bard College at Simon's Rock, Great Barrington, MA 01230, USA

**Keywords:** co-infection, disease ecology, *Gopherus*, *Mycoplasma*, quantitative PCR, upper respiratory tract disease

## Abstract

In disease ecology, the host immune system interacts with environmental conditions and pathogen properties to affect the impact of disease on the host. Within the host, pathogens may interact to facilitate or inhibit each other's growth, and pathogens interact with different hosts differently. We investigated co-infection of two *Mycoplasma* and the association of infection with clinical signs of upper respiratory tract disease in four congeneric tortoise host species (*Gopherus*) in the United States to detect differences in infection risk and disease dynamics in these hosts. Mojave Desert tortoises had greater prevalence of *Mycoplasma agassizii* than Texas tortoises and gopher tortoises, while there were no differences in *Mycoplasma testudineum* prevalence among host species. In some host species, the presence of each pathogen influenced the infection intensity of the other; hence, these two mycoplasmas interact differently within different hosts, and our results may indicate facilitation of these bacteria. Neither infection nor co-infection was associated with clinical signs of disease, which tend to fluctuate across time. From *M. agassizii* DNA sequences, we detected no meaningful differentiation of haplotypes among hosts. Experimental inoculation studies and recurrent resampling of wild individuals could help to decipher the underlying mechanisms of disease dynamics in this system.

## 1. Introduction

The manifestation of disease depends on interactions between the host and pathogen, in relation to the environment of the host. Pathogens can directly or indirectly affect other pathogens when co-infecting a host, and these microbial interactions can, in themselves, positively or negatively affect the survival of each pathogen within the host (e.g. [1]). Mutualistic, commensalistic or neutral interactions of pathogens within a host can result in additive or synergistic rates of clinical signs of disease. Alternatively, co-infection of pathogens that interact with each other negatively could result in disease with lower impact than what is expected additively.

Species in the tortoise genus *Gopherus* range from the Mojave Desert in the western United States to the longleaf pine forests of the southeastern USA, with different tortoise species inhabiting distinct vegetative ecosystems and climates. Four *Gopherus* spp. are found in the United States: Mojave Desert tortoises, *Gopherus agassizii*; Sonoran Desert tortoises, *Gopherus morafkai*; Texas tortoises, *Gopherus berlandieri*; and gopher tortoises, *Gopherus polyphemus*, which are broadly distributed in the southeastern USA. Additionally, multiple pathogens are known to be associated with an upper respiratory tract disease (URTD) in *Gopherus*, including *Mycoplasma agassizii*, *Mycoplasma testudineum*, *Pasteurella testudinis*, an iridovirus, and Testudinid herpesvirus 2 [2–7]. Thus, there is potential for different biological interactions among hosts and pathogens in the different ecosystems inhabited by different host species. Previous studies have detected URTD, and pathogens known to cause this disease, in each of the four tortoise species found in the USA [8–12]. Nevertheless, the majority of research on URTD has focused on *M. agassizii*.

Population declines in Mojave and gopher tortoises have been hypothesized to be caused by URTD, although the actual impacts of this disease to individuals and populations remains controversial [13,14]. Understanding disease dynamics in this system is especially important as both the Mojave Desert tortoise [15] and the gopher tortoise in the western portion of its range [16] are listed as threatened under the Endangered Species Act of 1973.

Here, we report on the prevalence of two pathogens in the tortoise-URTD system (*M. agassizii* and *M. testudineum*) in the four tortoise species within the USA to establish whether the occurrence or co-infection of these pathogens affects the manifestation of clinical signs of disease differently among hosts. We use nasal flushes to sample nasal microbes and visual observations of clinical signs of disease to answer: (i) are there genetically different bacterial types of the pathogen *M. agassizii* among tortoise species? (ii) at what frequencies are the pathogens *M. agassizii* and *M. testudineum*, both individually and in co-infection, present in local tortoise populations among tortoise species? and (iii) does the presence or co-infection of these pathogens predict the presence of clinical signs of disease?

## 2. Material and methods

### 2.1. Sampling and DNA extraction

**Figure 1.**
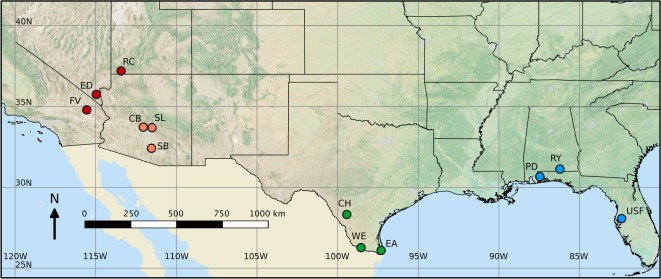
Sites sampled representing three geographical locations for each of four tortoise species: red = *Gopherus agassizii*, Mojave Desert tortoises; orange = *G. morafkai*, Sonoran Desert tortoises; green = *G. berlandieri*, Texas tortoises; blue = *G. polyphemus*, gopher tortoises. FV, Fenner Valley; ED, Eldorado Valley; RC, Red Cliffs; CB, Cave Buttes; SL, Sugarloaf; SB, Silverbell; CH, Chaparral Wildlife Management Area; WE, West Rio Grande; EA, East Rio Grande; PD, Perdido River; RY, Rayonier; USF, University of South Florida.

We used a 3 ml saline (0.9% NaCl) nasal lavage [17,18] to sample upper respiratory *Mycoplasma* from four species of wild-caught *Gopherus* tortoises (figure 1). Nasal lavage samples were immediately preserved in RNAlater stabilization solution (Ambion Inc., Austin, TX, USA) at a ratio of one part sample to five parts preservative volume. Samples were placed on ice in the field and frozen within 12 h. Each tortoise was sampled once, providing a snapshot of disease and pathogen patterns. All tortoises were captured by hand, except for gopher tortoises, which generally were trapped either with a pitfall trap or other live trap. While handling each tortoise, we recorded midline carapace length (MCL) and clinical signs of URTD. Clinical signs were scored based on severity: 0 = no signs; 1 = wheezing breath and/or damaged scales around nares; 2 = occluded nares; 3 = serous discharge from nares; 4 = purulent discharge from nares; 5 = nasal exudate and deeply damaged nares; 6 = nasal exudate and poor body condition [19]. Data on clinical signs were not available for 13 sampled Texas tortoises. We used secondary sex characteristics [20,21] to determine the sex of sampled tortoises in all adults. Juveniles were classified as animals without clear secondary sex characteristics and were, on average, 40–60% the average adult size.

Using the Qiagen DNeasy Blood and Tissue Kit (Qiagen Inc., Valencia, CA, USA) protocol for gram-negative bacteria, we extracted DNA from 500 µl of preserved lavage sample. We conducted polymerase chain reaction (PCR) for three genetic markers of *M. agassizii* (16S ribosomal RNA (rRNA), 16S-23S intergenic spacer region (IGS) and RNA polymerase beta subunit (rpoB)) [18] to detect genetic variation of this pathogen among tortoise species. PCR products of appropriate length were extracted from agarose gel using the Qiagen QIAquick Gel Extraction Kit and sequenced at the Nevada Genomics Center with an ABI3730 DNA Analyser (Applied Biosystems, Foster City, CA, USA). Sequences were identified as *Mycoplasma* using NCBI's BLAST search (http://blast.ncbi.nlm.nih.gov).

### 2.2. Differentiation of *Mycoplasma agassizii* among *Gopherus* spp.

A TCS haplotype network [22] was made for each of the three genetic markers from amplified sequences using PopArt [23]. Sequences were aligned in ClustalX 2.1 [24] and trimmed in MEGA6 [25]. Because sequences were of varying length, haplotype networks only included sequences at least 75% of the average length for each genetic marker. *Mycoplasma agassizii* sequences from GenBank (http://www.ncbi.nlm.nih.gov) were included in haplotype networks for all markers (type-strain PS6 [26] from a *G. agassizii* individual: accession numbers U09786.1, AY780802.1, EU925153.1). Additional laboratory sequences from the ATCC strain 723 cultured from a *G. polyphemus* were included in the haplotype networks for 16S rRNA and IGS.

### 2.3. Infection and co-infection analyses

We used a multiplex quantitative PCR (qPCR) protocol for *M. agassizii* and *M. testudineum* to detect both pathogens [27]. qPCRs were repeated in triplicate for each sample with thresholds determined by serial dilution curves. Samples that amplified in at least two reactions with a Cq value under 40 were considered positive for either pathogen of interest [27].

We used generalized linear models to detect differences in prevalence of *M. agassizii* and *M. testudineum* among tortoise species. Tukey's post hoc tests evaluated pairwise differences between tortoise host species. Analyses were run in the programming language R using the multcomp package for post hoc analyses (v. 3.2.1; [28,29]).

Samples were assigned the following infection codes based on qPCR results: 0 = neither *M. agassizii* nor *M. testudineum* detected, 1 = positive for *M. agassizii* only, 2 = positive for *M. testudineum* only, or 3 = positive for both *Mycoplasma* spp.

We used the multinom function in the nnet package [30] to conduct multinomial logistic regressions of sex and species or site to predict infection codes and Tukey's post hoc tests to evaluate differences among groups in the lsmeans package [31]. We corrected for the error rate owing to false discovery in multinomial logistic regression post hoc tests using the Benjamini Hochberg method, with a false discovery rate of 0.05, allowing for a highest significant *p*-value of 0.0183 [32,33].

To help determine under what circumstances hosts contract disease, we used generalized linear models to detect whether host sex, site, and infection code significantly predict the presence of clinical signs for each host species. We used linear models to detect a correlation of infection intensity between the two *Mycoplasma* spp. (using Cq value as a proxy).

## 3. Results

**Figure 2.**
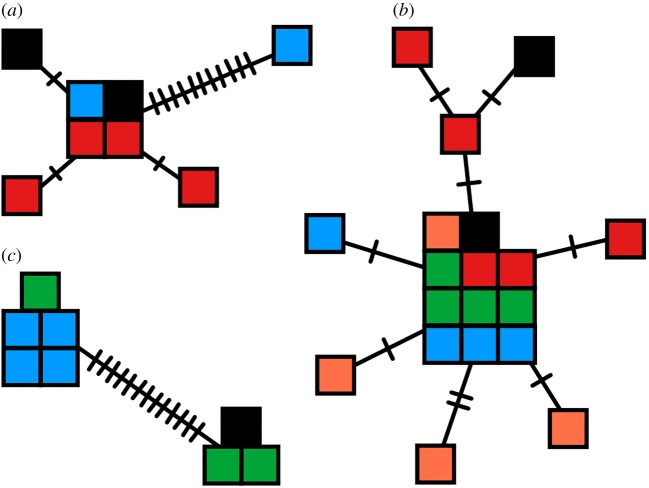
TCS haplotype networks of *Mycoplasma agassizii*. Colours based on host species or culture sequence: red = *Gopherus agassizii*, orange = *G. morafkai*, green = *G. berlandieri*, blue = *G. polyphemus*, black = culture sequences (from GenBank or in-lab sequencing). Each square represents a single sequence. (*a*) 16S ribosomal RNA with trimmed sequences 468 bp in length. GenBank sequence for type-strain PS6 split from the main haplotype, while the in-lab sequence for ATCC strain 723 from a gopher tortoise grouped with the main haplotype. (*b*) 16S-23S intergenic spacer region, sequences 476 bp in length. GenBank sequence for PS6 grouped with the core haplotype, while the in-lab sequence for ATCC strain 723 separated from the core haplotype by two single nucleotide polymorphisms.\break (*c*) RNA polymerase beta subunit sequences of 568 bp in length.

We sampled 256 tortoises between 2010 and 2015, including three geographical sampling sites for each species (table 1, figure 1). We used PCR with Sanger sequencing to detect genetic diversity of three markers in the *M. agassizii* genome, and we detected *M. agassizii* in 27 samples using this method (16S rRNA: *n* = 9; 16S-23S intergenic spacer: *n* = 19; rpoB: *n* = 9). We produced TCS haplotype networks from these data (figure 2), which did not suggest clear differentiation of *M. agassizii* populations among tortoise host species. Sequence data for 16S rRNA and rpoB were only successfully collected for two of the four tortoise host species sampled.

**Table 1.** Sample size in each *Gopherus* tortoise species and geographical sampling site, with relative proportion of each infection/co-infection result. (*Mycoplasma* spp. infections are values outside of co-infection. Results are from Tukey's post hoc tests of multinomial logistic regressions at a significant probability level cut-off of 0.0183. In the multinomial logistic regression, site was a significant predictor of infection, while species and sex were not (see text). Letters next to proportions denote significant differences within species or site.)

species, site	*n*	no pathogen detected		*M. agassizii*		*M. testudineum*		co-infection	
*G. agassizii*	57	0.28	a	0.44	a	0.04	b	0.25	a
Eldorado	17	0.12	ab	0.18	ab	0.06	a	0.65	b
Fenner	19	0.16	ab	0.74	a	0.05	b	0.05	b
Red Cliffs	21	0.52	a	0.38	ab	0.00	b	0.10	ab
*G. morafkai*	51	0.53	a	0.25	b	0.02	b	0.20	b
Cave Buttes	8	0.50	a	0.13	b	0.13	b	0.25	ab
Silverbell	18	1.00	a	0.00	b	0.00	b	0.00	b
Sugarloaf	25	0.20	ab	0.48	a	0.00	b	0.32	ab
*G. berlandieri*	56	0.68	a	0.00		0.23	b	0.09	b
Chaparral WMA	30	0.63	a	0.00		0.37	a	0.00	b
East Rio Grande	13	0.62	a	0.00		0.08	a	0.31	a
West Rio Grande	13	0.85	a	0.00		0.08	b	0.08	b
*G. polyphemus*	92	0.64	a	0.14	b	0.09	b	0.13	b
Perdido	29	0.69	a	0.24	b	0.00	b	0.07	b
Rayonier	32	0.69	a	0.03	b	0.19	b	0.09	b
Univ. South Florida	31	0.55	a	0.16	b	0.06	b	0.23	ab

**Figure 3.**
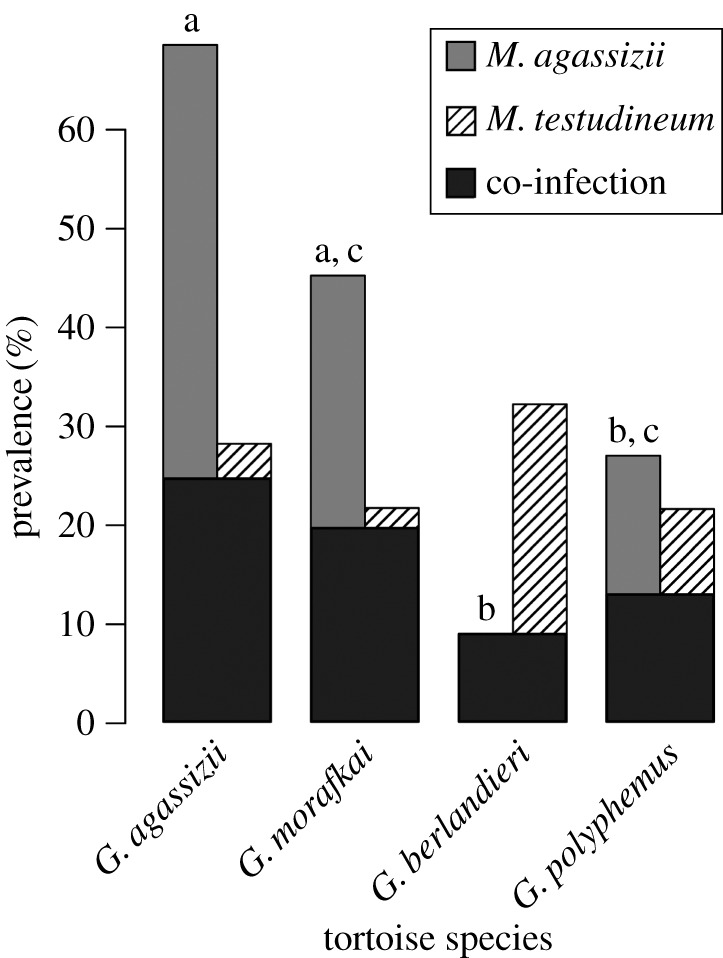
Barplot of prevalence of *Mycoplasma agassizii* (light grey), *M. testudineum* (stripes) and co-infection of the two *Mycoplasma* spp. (dark grey) by host species. Letters above *M. agassizii* bars denote significant differences in prevalence of that pathogen between the host species from Tukey's post hoc tests on a generalized linear model at a significance of 0.05.

Using a probe-based qPCR assay [27], we detected *M. agassizii* in 92 samples and *M. testudineum* in 65 samples, ranging in prevalence across the tortoise host species (figure 3). In generalized linear models, host species differed in prevalence of *M. agassizii*: desert tortoise species had higher prevalence than Texas tortoises (*p* < 0.001 each) and Mojave Desert tortoises had higher prevalence than gopher tortoises (*z *= –4.768, *p* < 0.001). Prevalence of *M. testudineum* did not differ by host species (*p *> 0.4 each; figure 3). In Texas tortoises, every individual with detectable *M. agassizii* was also co-infected with *M. testudineum* (figure 3). Both *Mycoplasma* spp. were detected in males, females, and juveniles in all four tortoise host species, except juvenile Sonoran Desert tortoises (*n* = 2), in which we did not detect either pathogen. Infection load of each pathogen was generally relatively low, represented by high Cq values (average Cq of 37.5 ± 1.52 (s.d.) for *M. agassizii*, 37.2 ± 1.80 for *M. testudineum*), and Cq values ranged from 30.9 to 39.9, representing an approximately 200- to 500-fold increase (depending on the pathogen) from the lowest to the highest load detected. Of the samples positive for *Mycoplasma*, only six (*M. testudineum*) or seven (*M. agassizii*) samples had a Cq value under 35.

For the following analyses, tortoise samples were categorized into four infection groups from qPCR results: 0 = neither *Mycoplasma* detected; 1 = *M. agassizii* detected; 2 = *M. testudineum* detected; 3 = both *Mycoplasma* spp. detected. The presence of these infection groups was analysed using multinomial logistic regressions. Model results indicated that species (all data) and sex (all data and separated by species) were not significant predictors of infection group (*p > *0.4 each). Site was a significant predictor in the all-inclusive model (*χ*^2^_33_ = 100.58, *p* < 0.0001) and in individual species models (*p* ≤ 0.008 each). Below, we present results of post hoc tests within and among sites of each tortoise host species sampled.

### 3.1. Mojave Desert tortoise infection

The number of Mojave Desert tortoise samples either uninfected or infected with just *M. agassizii* did not significantly differ, while significantly fewer samples had a single infection of *M. testudineum* or co-infection of both species of *Mycoplasma* (table 1). Tortoises from Eldorado Valley had lower prevalence of *M. testudineum* in sole infection versus co-infection. More individuals in Fenner Valley had a sole infection of *M. agassizii* than *M. testudineum* or co-infection of both mycoplasmas. In Red Cliffs tortoises, lack of infection was more frequent than sole infection with *M. testudineum*. The three sampled tortoise populations in the Mojave Desert differed from each other in infection-group patterning (*p* < 0.009 each, pairwise).

### 3.2. Sonoran Desert tortoise infection

Regarding infection in Sonoran Desert tortoises, more individuals were uninfected by either *Mycoplasma* than infected with one or both pathogens. In Cave Buttes, prevalence of either pathogen in sole infection was lower than the absence of infection (table 1). Infection prevalence of any type was significantly lower than frequency of no infection in Silverbell tortoises. In Sugarloaf tortoises, there was a difference in prevalence of sole infection between the two *Mycoplasma* spp., with higher prevalence of *M. agassizii*. Among sampling sites, infection status in Sugarloaf and Cave Buttes samples significantly differed from the infection-group pattern in Silverbell tortoises (*p* < 0.0001 each), though Sugarloaf and Cave Buttes infection patterns did not differ from each other (*F*_3,15_ = 1.684, *p* = 0.21).

### 3.3. Texas tortoise infection

In Texas tortoises, no individuals had *M. agassizii* as a sole infection, and the absence of any infection was more common than the presence of *M. testudineum* or co-infection (table 1). At Chaparral Wildlife Management Area, no samples were positive for *M. agassizii*, and thus no individuals had co-infection of both *Mycoplasma* spp., but the presence and absence of *M. testudineum* occurred at similar rates. Tortoises from East Rio Grande had *M. testudineum* infection less frequently than no infection, and at West Rio Grande, the absence of infection was more frequent than either *M. testudineum* infection or co-infection (table 1). Samples from the three sites representing Texas tortoises did not significantly differ from each other in infection-group patterning (*p* > 0.25 each).

### 3.4. Gopher tortoise infection

Pairwise comparisons in gopher tortoises revealed significantly more individuals with an absence of infection than any other infection status (table 1). At Perdido and Rayonier sites, the rate of no infection was greater than any other infection group. In Perdido tortoises, prevalence of *M. agassizii* in sole infection was higher than prevalence of *M. testudineum*. At the University of South Florida site, the absence of infection was more frequent than sole infection with either *Mycoplasma*, but not more frequent than co-infection (table 1). Samples from the three sites representing gopher tortoises did not significantly differ from each other in infection-group patterning (*p* > 0.18 each).

### 3.5. Clinical signs of disease

**Figure 4.**
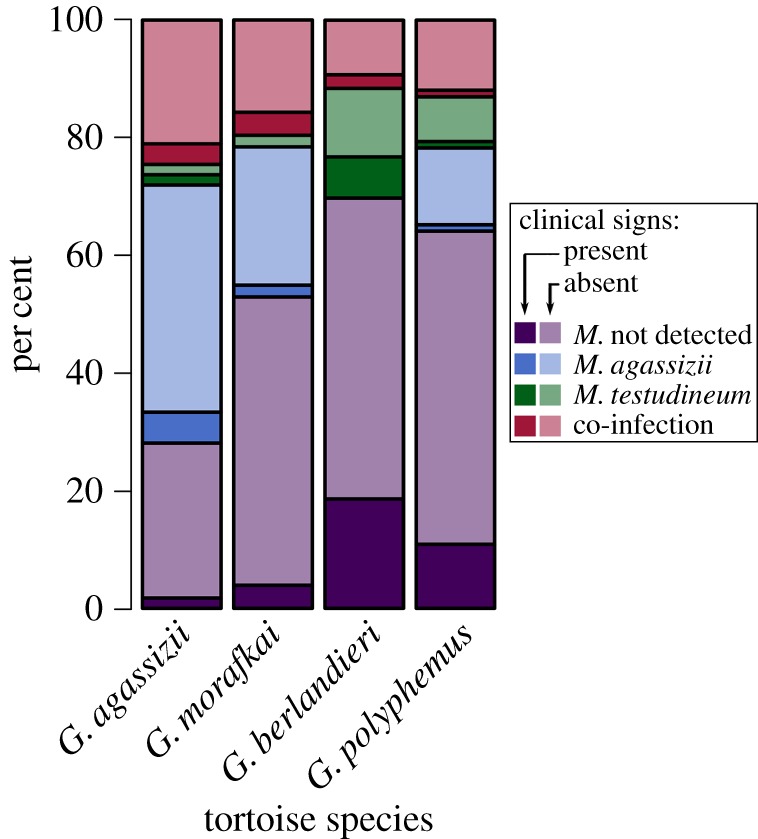
Stacked bar plot of infection groups split by the presence of clinical signs of disease in each *Gopherus* tortoise host species. Colours denote infection group, with lighter shades indicating absence of clinical signs of disease: purple = no *Mycoplasma* pathogen detected, blue = *M. agassizii* sole infection, green = *M. testudineum* sole infection, red = co-infection of both *Mycoplasma*. In *G. berlandieri*, 13 tortoises were not evaluated for clinical signs and are thus excluded from the total.

From generalized linear models, none of the infection groups significantly predicted the presence of clinical signs of URTD for any host species, and most of the tortoises with clinical signs of disease did not have detectable amounts of either *M. agassizii* or *M. testudineum* in their upper respiratory tracts (*p *> 0.1; figure 4). In gopher tortoises, we found that individuals from the University of South Florida site had significantly lower rates of clinical signs of URTD than Perdido tortoises (USF 3%, Perdido 28%, Tukey's post hoc, *z* = –2.408, *p* = 0.041).

Although we found clinical signs of disease in the form of nasal discharge or tissue damage from recent discharge in each of the four tortoise species, none of the Texas or Sonoran Desert tortoises sampled had nasal discharge at the time of sampling. Rather, clinical signs of disease in those species manifested as eroded scales around the nares or occluded nares (severity score 1–2). Serous nasal mucus (severity score 3) was found in gopher (*n* = 3) and Mojave Desert (*n* = 6) tortoises at the time of sampling. One additional gopher tortoise had severely damaged nares and purulent nasal mucus (severity score 5), and we did not detect either species of *Mycoplasma* in her lavage sample.

### 3.6. Inter-pathogen associations

**Figure 5.**
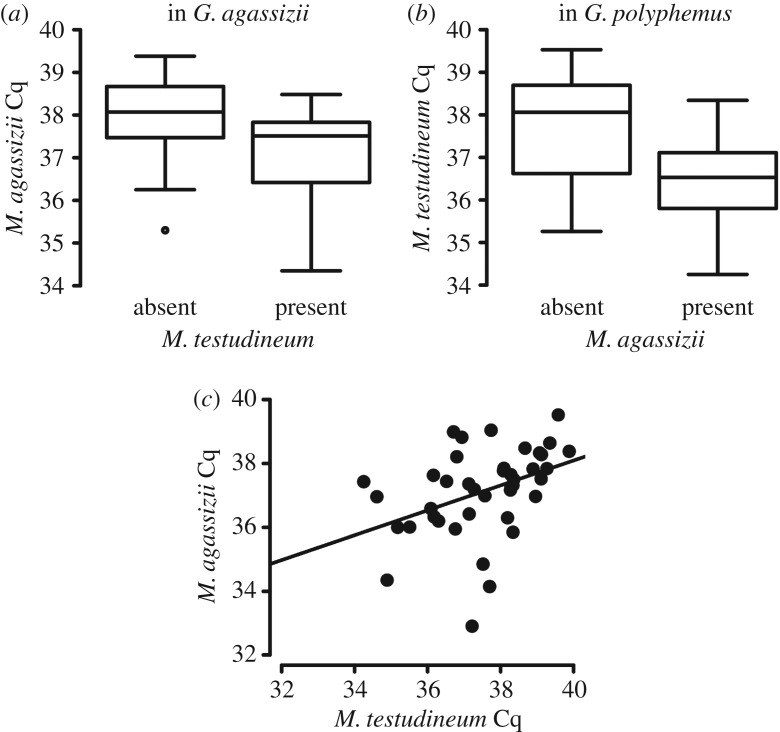
Presence and infection intensity (Cq as a proxy) of each *Mycoplasma* species based on the other. Lower Cq values indicate higher microbial load in the sample. (*a*) *Mycoplasma agassizii* with and without *M. testudineum* in *Gopherus agassizii* (Mojave Desert tortoises; *F*_1,37_ = 6.428, *p* = 0.016); (*b*) *M. testudineum* in samples with and without *M. agassizii* in *G. polyphemus* (gopher tortoises; *F*_1,18_ = 4.447, *p *= 0.049); (*c*) correlation of Cq values between the two *Mycoplasma* spp. assayed (*R*^2^ = 0.159, *F*_1,39_ = 7.39, *p *= 0.0097).

A greater *M. agassizii* load (lower Cq value) was correlated with the presence of *M. testudineum* in tortoise nasal lavage samples (*F*_1,90_ = 5.49, *p* = 0.02). This relationship was probably driven by its significance in the Mojave Desert tortoise (*F*_1,37_ = 6.428, *p *= 0.016; figure 5*a*). Because all Texas tortoises with detectable *M. agassizii* also had *M. testudineum*, we could not compare differences in *M. agassizii* load between the presence and absence of *M. testudineum*.

Within all host species combined, the presence of *M. agassizii* in nasal lavage samples was marginally associated with less *M. testudineum* (*F*_1,63_ = 3.859, *p* = 0.054), though in gopher tortoises, more *M. testudineum* was correlated with the presence of *M. agassizii* (*F*_1,18_ = 4.447, *p *= 0.049; figure 5*b*).

Finally, an increase in amount of either bacterium correlated with an increase in amount of the other species (*F*_1,39_ = 7.39, *p *= 0.0097; figure 5*c*). However, no host species followed this pattern without it being driven by a single, strongly infected sample.

## 4. Discussion

We detected both *M. agassizii* and *M. testudineum* in all four tortoise species inhabiting the USA. These pathogens have been reported to cause URTD in tortoise hosts, and some have hypothesized that URTD has caused population declines in Mojave Desert tortoises and gopher tortoises [4,5,14,34–36]. We aimed to decipher the evolutionary history of this pathogen and detect the bacterial strain diversity within and among host populations with sequences of three genetic markers of *M. agassizii*. Multiple strains of *M. agassizii* have been found in Mojave Desert tortoises [26], but no efforts have since been made to determine the variation of *Mycoplasma* diversity within and among hosts. If some strains are more associated with disease than others, then relevant host populations could be appropriately managed. However, we did not detect any meaningful differentiation of this pathogen among its tortoise hosts. Because our current understanding of the *M. agassizii* genome lies in its conserved regions, we suggest that genomic data of these bacteria would allow us to determine bacterial strain diversity within and among host populations, and potentially to discover the relative virulence of strains. However, variable virulence can also occur with minimal genetic diversity [37]. Even clonal mycoplasmas can present high diversity of surface antigens through phenotypic plasticity [37]. Infection of mycoplasmas by viral phages can also impact the bacteria's pathogenicity [37]. Furthermore, diversity of tandem repeat sections of the genome can allow for increased chromosomal rearrangement and phase variation in the cell surface antigens of *Mycoplasma* [37].

Thus far, the focus of research regarding URTD has been on the pathogen *M. agassizii*, even though URTD in tortoises is also associated with *M. testudineum*, *P. testudinis*, an iridovirus, and Testudinid herpesvirus 2 [2,3,5–7]. We assayed tortoise samples for both *M. agassizii* and *M. testudineum*, and our results did not detect an infection or co-infection status that best associated with the presence of clinical signs of disease (figure 4). In disease systems, there is an incubation period between colonization of the nasal passages and the onset of clinical signs (and the ability to transmit pathogens). Frequently, there is an assumption that the presence of clinical signs of disease, such as nasal discharge, is associated with shedding of bacteria, enabling disease spread; more often than not, we were unable to detect either *Mycoplasma* in samples collected from tortoises with clinical signs of disease (figure 4). Half (5 out of 10) of the tortoises exhibiting nasal discharge at the time of sampling did not have detectable amounts of either pathogen in their lavage samples. Thus, while these pathogens or their interactions with yet additional pathogens may be the cause of disease, the presence of nasal discharge may be a necessary, but clearly not sufficient, variable in the spread of *Mycoplasma*. Importantly, nasal discharge could be caused by a variety of factors, not all of which are disease-related. As such, the presence of discharge should not be assumed to indicate an infection by pathogens.

Little is known about the prevalence or progression of infections in juvenile *Gopherus* tortoises. Recent research by Aiello *et al*. [38] found that, on average, long periods of direct nose-to-nose contact are required for *M. agassizii* to be transmitted between adult Mojave Desert tortoises. It seems that burrow sharing at night is the most likely means of exposure, and reproductive tortoises are the most likely individuals to interact with conspecifics via burrow sharing or using multiple different burrows [39]. Previous research has reported disease in subadults, even though its spread is associated with social interactions attributed to adults [40,41]. We included pre-reproductive tortoises in the present study and found infection by both mycoplasmal pathogens in young individuals of each host species except Sonoran Desert tortoises. Though vertical transmission cannot be ruled out, research has shown that vertical transmission is unlikely for *M. agassizii* [42] and has not been studied for *M. testudineum*. *Gopherus* can reach sexual maturity in their teens [43], and thus they have many years to interact with others before the onset of social interactions associated with mating. Juvenile desert tortoises do occasionally share burrows with adult tortoises [44], as do juvenile gopher tortoises with other juveniles [45], and the extremely low likelihood of *Mycoplasma* surviving in burrow soil [46] suggests that direct contact is required for pathogen transmission. A recent study found that aggressive behaviours lead to contact between juveniles [45]. Additional research examining pre-reproductive behaviours of tortoises that allow them to become infected would greatly help in our understanding of transmission of pathogens associated with URTD. Importantly, we suspect that these *Mycoplasma* species, as in other systems, may have life-long, chronic, cyclical interactions with their tortoise hosts [18]. If this is the case, the rate of infection in young tortoises may be an important factor in the prevalence and dynamics of these pathogens among local host populations.

Our analyses suggest that Mojave Desert tortoises are more likely than other tortoise species to have detectable amounts of *M. agassizii* in their upper respiratory tracts. Local populations are in decline across most of the Mojave Desert tortoise distribution [47], and understanding threats by pathogens is important for management initiatives. When tortoises are translocated as part of management schemes, knowing the pathogen prevalence in the source and destination populations could be critically important in predicting survival of local populations and ensuring translocation success. Although this disease has been hypothesized to cause population declines via host mortality, it seems more likely that under normal conditions, infected tortoises will be minimally harmed, with a chronic latent infection [14]. However, if disease severity increases as a consequence of environmental stressors, then factors such as habitat alterations and a changing climate could compound to influence the disease trajectory in hosts, as is the case in other systems [48]. While translocation might not directly act as a stressor to tortoise individuals [49], the increase in local population density owing to translocations, coupled with increased connectivity of local populations from the movement of translocated animals, could lead to increased interactions among tortoises and thus increased transmission of pathogens [50]. Importantly, pathogens present in both the source and destination populations could have increased transmission from host translocations, as an influx in susceptible individuals supports pathogen spread [51].

*Gopherus* tortoises can live for up to 80 years, and individuals reproduce throughout adulthood, with low adult mortality rates [15]. Some local populations are probably more at risk because of disease than others [52]. For the long-term management of Mojave Desert tortoises, focus should be placed on minimizing stressors to decrease the risk of exacerbating the severity of URTD, as stronger infection loads have been linked to higher risk of transmission [38]. URTD can also lower the reproductive success of adult females, and high prevalence of URTD in reproductive females might significantly reduce recruitment, contributing to tortoise population declines [53].

Interestingly, while we detected mild clinical signs of disease in Sonoran and Texas tortoises, none of these tortoises examined exhibited mucus discharge. This suggests that mycoplasmal pathogens might interact differently with different host species. Variance in disease dynamics among host populations occurs in other host–pathogen systems, such as with the fungus *Batrachochytrium dendrobatidis* (Bd) in frog species. Among frog species, some experience very high morbidity and mortality rates owing to chytridiomycosis, the disease caused by Bd, while others are carriers, unaffected by the pathogen, yet they are able to spread it [54]. Species that experience morbidity owing to chytridiomycosis do so along a spectrum of harm [55]. The four tortoise species in our study experience different ecological interactions in different ecosystems, so we should not assume that pathogens affect these host species similarly. In the Texas tortoise, *M. agassizii* was only detected in co-infection with *M. testudineum*. Future research should determine whether or not this pattern persists within larger sample sizes and at different sites within the Texas tortoise distribution. None of the Texas tortoises sampled (*n* = 56) had nasal discharge present at the time of sampling, even though URTD has been detected in this species, particularly in captive individuals [9]. As most research on this disease has focused on gopher and Mojave Desert tortoises, further studies should determine how pathogens that cause URTD in other tortoise hosts affect species for which this disease system is less well understood.

The interactions between pathogens within a host could be facilitative, competitive, antagonistic or neutral. Telfer *et al.* [1] investigated the range of interactions among four pathogens infecting locations throughout a vole host species and found a wide range of positive and negative interactions. The type and strength of interactions were not necessarily reciprocal and depended on recency and whether an infection was acute or chronic. In our data, while the presence of each pathogen affected the infection intensity of the other (figure 5), this pattern was not consistent across host species. These results indicate that the two pathogens might facilitate each other in a context-dependent manner, and that these pathogens interact differently with their hosts and each other under differing conditions. These hypotheses should be further investigated.

Here, we provide a snapshot view of *Mycoplasma* prevalence in a few local populations of four North American tortoise species. The scope of this research constrained us to choosing three distinct sampling locations to represent entire host species, so the extent to which this data may be extrapolated should be considered with caution. Each of these tortoise species' distributions cover wide ranges of abiotic environmental variables and variation in vegetative land cover. As such, the similarities and differences among the sampling sites detected in our data can be used to discuss the possibilities of what may be occurring elsewhere in these species' ranges. Furthermore, the patterns found in our data are probably affected by other microbes present in the upper respiratory tract. While we have data on co-infection rates between *Mycoplasma* spp., we still lack prevalence data on other associated pathogens in the system. From preliminary pyrosequencing data, we have found *P. testudinis* in 50–92% of samples tested from the same four tortoise species (C. L. Weitzman 2017, unpublished data from a microbiome study). A probe-based qPCR approach to assay for *P. testudinis* would enable us to add a piece to the puzzle to best determine how pathogens interact to cause URTD.

In our study system, two *Mycoplasma* pathogens are present in differing co-infection patterns depending on the host species. Our data suggest a low amount of genetic variation in *M. agassizii* among four allopatric host species that span the two coasts of the United States. In addition, a majority of the tortoises sampled with clinical signs of URTD did not have detectable amounts of either *Mycoplasma* in their nasal lavage samples. The two species with lower pathogen prevalence, gopher tortoises and Texas tortoises, had greater prevalence of clinical signs than the other species sampled (figures 3 and 4). These results suggest that long-term data, assaying for additional pathogens, and monitoring environmental stressors might be necessary to understand more fully the role of each pathogen type in the cause and severity of disease. Recurrent resampling of individuals and assaying for all known pathogens would provide a more thorough view of pathogen dynamics over time, and an artificial experiment study on co-infections would disentangle how these pathogens interact within their hosts.

## Supplementary Material

Host, Infection, and Clinical Signs Data

## Data accessibility

DNA sequences are available on GenBank, accessions MF966905-MF966920. The remaining data supporting this article have been uploaded as the electronic supplementary material.
